# Dynamics of petal pigments and morpho-physiological traits during flower development in *Rosa* ‘Creamy Eternity’

**DOI:** 10.7717/peerj.21329

**Published:** 2026-08-06

**Authors:** Zilong Ouyang, Tian Xu, Quanguang Yang, Liwen Mo, Xianglu Jia, Xiaoyu Gao, Miaoqin Wei, Lurong Pan, Tinghui Yin, Hongying Liao, Qiangyu Long, Lei Zhang, Weichao Teng

**Affiliations:** 1 Nanning Botanical Garden, Nanning, China; 2 Nanning Qingxiu Mountain Scenic Area Tourism Development Co., Ltd., Nanning, China; 3 Nanning Wuxiangling Forest Park, Nanning, China; 4 Nanning Horticultural Expo Garden Administration Center, Nanning, China; 5 Guangxi Eco-Engineering Vocational & Technical College, Liuzhou, China; 6 Guangxi University, Nanning, China

**Keywords:** *Rosa* ‘Creamy Eternity’, Flower development, Petal, Pigment, Morphological structure, Physiological

## Abstract

As an important ornamental plant, rose (*Rosa* spp.) undergoes dynamic changes during flowering development, involving multiple indicators such as pigment composition, morphological structure, nutrient content, antioxidant capacity, and mineral element levels. However, the dynamic patterns and interactions among these physiological and biochemical indicators throughout flowering remain inadequately characterized. In this study, *Rosa* ‘Creamy Eternity’ was used as plant material, and five distinct developmental stages were examined: flower bud stage (S1), bud emergence stage (S2), bud swelling stage (S3), first bloom stage (S4), and full bloom stage (S5). Comprehensive analyses were conducted to assess dynamic changes in color parameters, pigment content, morphological structure, nutrient content, antioxidant capacity, and mineral element composition. The results revealed that: (1) During flower development, chlorophyll (Chl) content decreased markedly, while carotenoid (Car) and anthocyanin (AC) contents also declined. In contrast, flavonoid (Fla) content increased at stage S3, contributing to color stability. Petal color shifted from yellow-green to light yellow, accompanied by an increase in a* and L* values and a decrease in b* value; (2) cellular expansion and tissue development progressively refined petal morphology, increasing internal air space volume and petal thickness (PT). The upper epidermal cells transitioned from conical to flattened shapes, thereby enhancing light reflectance and overall brightness; (3) non-structural carbohydrates (NSC) accumulated during early flowering, after which starch (St) was broken down into soluble sugar (SS) to sustain energy metabolism. Soluble protein (SP) content increased continuously, reaching its peak at full bloom; (4) malondialdehyde (MDA) accumulation during early flowering likely activated the antioxidant enzyme system, which acted synergistically to alleviate oxidative damage by reducing MDA levels; (5) mineral elements exhibited significant correlations and functioned synergistically in regulating nutrient partitioning across flowering stages, with distinct elemental requirements at different phases; (6) stage S4 was identified as a critical period for floral organ development. In summary, optimized fertilization management during early flowering is recommended to enhance ornamental performance at full bloom. These findings provide a theoretical foundation for improved cultivation and flowering regulation in *Rosa* ‘Creamy Eternity’.

## Introduction

The ornamental value of flowering plants is largely determined by their floral traits (*e.g.*, color variation, morphological structure), which undergo dynamic changes throughout the flowering process—from the flower bud stage to full bloom. This process is co-regulated by multiple factors, including nutrient levels, stress resistance, and mineral element content. Petal coloration is primarily governed by pigments such as chlorophyll (Chl), anthocyanins (AC), flavonoids (Fla), and carotenoids (Car) (Tanaka, Sasakia & Ohmiya, 2008). Chl imparts a green color (Yuan et al., 2025), while carotenoids and flavonoids are responsible for yellow to orange hues (Cruz et al., 2024; Liu et al., 2020). AC appear blue or bluish-purple in alkaline conditions and red in acidic environments (Liu et al., 2025). Changes in pigment composition during flowering lead to color shifts, directly influencing petal coloration. The morphological structure of petals further modulates color appearance through its physical and optical properties. The thickness-to-width ratio (TWR) of the upper epidermal cells determines their shape: conical cells with a higher TWR enhance light refraction and absorption, deepening petal color, whereas flattened cells with a lower TWR promote light reflection, resulting in a lighter appearance (Lee et al., 2011). Petal color can be quantitatively described using color parameters (*L*^∗^, *a*^∗^, *b*^∗^, *C*^∗^, *h*^∗^) (Sendri et al., 2024; Yin et al., 2022). For instance, in *Red raspberry, L*^∗^, *a*^∗^ and *b*^∗^ values show significant correlations with total anthocyanin content (Baldassi et al., 2024). Similarly, these parameters have been used to evaluate traits in *Fragaria chiloensis* and *Chrysanthemum indicum* germplasm (Li et al., 2023b; Mora, Zúñiga & Figueroa, 2019).

Non-structural carbohydrate (NSC), starch (St), soluble sugar (SS), and soluble protein (SP) are essential nutrients that not only provide energy and substrates for key developmental steps but also indirectly participate in color formation and flower longevity (Bao et al., 2020; Shi et al., 2024). Antioxidant enzymes including superoxide dismutase (SOD), peroxidase (POD), catalase (CAT), and ascorbate peroxidase (APX) play crucial roles in stress response and senescence by collaboratively converting superoxide anions and reactive oxygen species (ROS) into hydrogen peroxide (H_2_O_2_) and oxygen (O_2_), thereby mitigating oxidative damage (Kaur, Singh & Mukherjee, 2014). Their activities fluctuate dynamically during flowering, increasing significantly under stress to reduce malondialdehyde (MDA) accumulation and maintain membrane integrity and flowering stability (Bo et al., 2024; (Korkmaz, 2023). Nitrogen (N), phosphorus (P), potassium (K), calcium (Ca), magnesium (Mg), zinc (Zn), boron (B), iron (Fe), manganese (Mn), sulfur (S) are essential mineral elements for floral organ development and regulate both flowering timing and quality. They also indirectly influence coloration by affecting vacuolar pH, pigment-related enzyme activity, and AC stability through mechanisms such as metal complexation (Takeda, 2006; Wieland & Aarts, 1980; Akbari et al., 2012; Ellestad, 2006; Cho et al., 2025). Nutrient deficiencies can delay flowering and significantly impair floral quality (Johnson, 1973).

Rose (*Rosa* spp.), a typical flowering plant in the Rosaceae family, is widely cultivated globally and holds substantial market value (Cheng et al., 2025). Its versatile landscaping applications include open-field planting, potted cultivation, and cut flowers, suitable for various settings such as homes, parks, and roadsides. The ornamental traits and flower longevity of roses directly affect their landscape value and industrial development, making it essential to understand the flowering process (from the flower bud stage to full bloom), to maintain desirable traits, and extend the ornamental period. *Rosa* ‘Creamy Eternity’ is characterized by its long flowering period, strong disease resistance, and abundant blooms. Its cup-shaped flowers with creamy green petals that gradually lighten during flowering make it suitable for promotion in South China. Although previous studies have examined the physiological biochemistry, nutritional value, flowering regulation, and postharvest longevity of roses (Abdul et al., 2022; Sarkari et al., 2024; Rezaei et al., 2024; Stamin et al., 2024), the dynamic changes and synergistic regulation of color parameters, morphological structure, nutrients, antioxidants, and mineral elements during flowering in *Rosa* ‘Creamy Eternity’ remain insufficiently studied.

Therefore, this study investigates the dynamic changes in color (*L*^∗^, *a*^∗^, *b*^∗^, *C*^∗^, *h*^∗^, Chl, AC, Fla, Car), morphological structure (*e.g.*, TWR), nutrients (*e.g.*, NSC, St, SS, SP), antioxidant capacity (*e.g.*, SOD, POD, CAT, APX), and mineral elements (N, P, K, Ca, Mg, Zn, B, Fe, Mn, S) during the flowering of *Rosa* ‘Creamy Eternity’. We aim to characterize the dynamic patterns of color change, morphological development, physiological and biochemical responses, as well as to explore the synergistic interactions among these indicators, providing a theoretical basis for the cultivation management and flowering regulation of this cultivar.

## Materials and Methods

### Study site and species

The experiment was conducted at the Plant Germplasm Resource Nursery of Nanning Botanical Garden (22°47′50.14″N, 108°24′16.60″E), located in Guangxi, China. The region experiences a subtropical monsoon climate characterized by warm temperatures, abundant sunshine, and plentiful rainfall. The mean annual temperature is 21.8 °C, with an average annual sunshine duration of 1480.4 h and an average annual precipitation of 1,453.4 mm.

**Figure 1:**
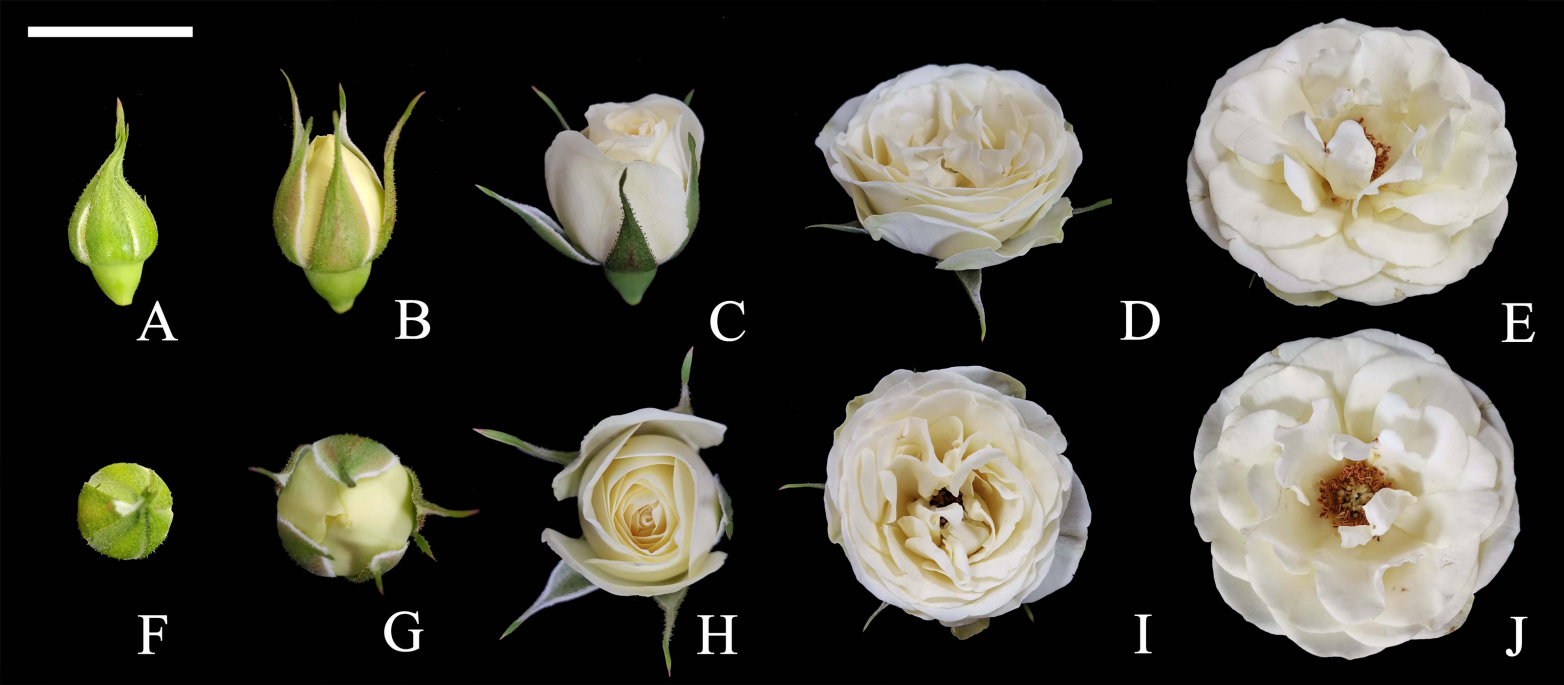
Photographs depicting the five flowering stages of *Rosa* ‘Creamy Eternity’. Flower bud stage (Stage 1, S1) (A, F), bud emergence stage (Stage 2, S2) (B, G), bud swelling stage (Stage 3, S3) (C, H), first bloom stage (Stage 4, S4) (D, I), full bloom stage (Stage 5, S5) (E, J). Scale bar = 3 cm.

The plant material used in this study was *Rosa* ‘Creamy Eternity’. *Rosa* ‘Creamy Eternity’ is a miniature Floribunda rose (Rosaceae) derived from ‘Million Yellow’ × ‘Sakura’. It produces cup-shaped, mildly fragrant flowers measuring approximately six cm in diameter when fully opened, with 25–30 petals per flower—a moderate petal count compared to Damask roses (*Rosa damascena*) (Omidi et al., 2022). The cultivar exhibits a shrub-like growth habit with recurrent blooming. Based on preliminary observations of its flowering process, flower development was divided into five stages: flower bud stage (S1), bud emergence stage (S2), bud swelling stage (S3), first bloom stage (S4), and full bloom stage (S5) (Fig. 1). A randomized block design was adopted, with three independent experimental plots serving as block units. Each plot contained 16 uniformly growing individuals of *Rosa* ‘Creamy Eternity’, totaling 48 plants across all three plots.

In mid-April 2025, which represents the peak flowering period of *Rosa* ‘Creamy Eternity’ in Nanning, Guangxi, petal samples were collected from the central region of middle-layer petals, avoiding both the base and the margin. For each developmental stage within the same block, petal samples were thoroughly mixed to form a composite sample, which was regarded as one biological replicate. Three biological replicates were obtained from the three experimental plots. All samples were immediately placed in ice boxes and transported to the laboratory for further processing. Collected samples were divided into four groups: (1) Samples for immediate measurement of color parameters and pigment content; (2) samples for morphological observation, fixed with FAA under vacuum infiltration and embedded in paraffin for sectioning; (3) samples for total carbon and mineral element analysis, dried to constant weight after enzyme inactivation at 100 °C for 30 min and subsequent drying at 70 °C for 10 h; (4) samples for antioxidant physiological indices and nutrient content determination, stored at −80 °C until analysis. All measurements were performed with three biological replicates, each consisting of three technical replicates. The temperature data for this month is provided in Supplemental Information 1.

### Determination of petal color parameters, pigments content and pH

Petal color attributes were quantified based on the CIE *L*^∗^*a*^∗^*b*^∗^ uniform color space (Gonnet, 1993). A spectrophotometer (DS-700E, Hangzhou CHNSpec Technology Co., Ltd, China) with a C/2° illumination setup (Hao et al., 2020) was employed to measure *L*^∗^ (lightness), *a*^∗^ (red-green value), *b*^∗^ (yellow-blue value), *C*^∗^ (chroma), and *h*^∗^ (hue angle). In this system, higher *L*^∗^ values indicate brighter petals; higher positive *a*^∗^ values correspond to stronger redness; higher positive *b*^∗^ values indicate stronger yellowness; higher *C*^∗^ values reflect more vivid or intense coloration; and *h*^∗^ (hue angle) defines the tonal angle, where values near 0 represent red and those approaching 90 represent yellow.

For pigment extraction, 0.2 g of fresh petal tissue from the central region was finely cut and incubated in five mL of 80% acetone at 4 °C under dark conditions until complete bleaching occurred. The resulting supernatant was collected by filtration. Chlorophyll (Chl) and carotenoid (Car) contents were determined according to established protocols using a full-wavelength microplate reader (Varioskan Flash3001, Thermo Fisher Scientific Inc, Germany) (Lichtenthaler & Buschmann, 2001; De Souza et al., 2023). Anthocyanin (AC) and flavonoid (Fla) levels were measured with commercial assay kits (Shanghai Win-win Biotechnology Inc.).

To determine petal pH, one g of fresh central petal tissue was homogenized in eight mL of deionized water, and the pH of the slurry was immediately recorded using a pH meter (PHS-3E; Shanghai Leici Scientific Instrument Co., Ltd., Shanghai, China) (Zhao et al., 2013).

### Determination of petal pigment-related and antioxidant enzyme activities and malondialdehyde content

Phenylalanine ammonia-lyase (PAL) activity was measured using a commercial kit (Shanghai Win-win Biotechnology Inc.).

For antioxidant enzyme assays, 0.3 g of fresh petal material was ground in liquid nitrogen with polyvinylpyrrolidone (PVP) and quartz sand, then homogenized in three mL of 0.05 mol L^−1^ phosphate buffer (pH 7.0). The homogenate was centrifuged at 11,000× g for 20 min at 4 °C, and the supernatant was collected as the crude enzyme extract. Activities of superoxide dismutase (SOD), peroxidase (POD), catalase (CAT), and ascorbate peroxidase (APX) were determined following published methods (Zhao et al., 2024; Zhang et al., 2024).

Malondialdehyde (MDA) content was assessed by homogenizing one g of petal tissue in 10% trichloroacetic acid, followed by centrifugation. The supernatant was used for MDA quantification according to the referenced procedure (Hidalgo et al., 2021).

### Determination of petal non-structured carbohydrates, total carbon and soluble protein content

Starch (St) and soluble sugar (SS) contents were analyzed as previously described (Zhou et al., 2009). Briefly, 0.2 g of frozen petal powder was extracted with 80% ethanol for SS determination using the anthrone method. The residue was further processed for St measurement *via* perchloric acid hydrolysis followed by anthrone assay. Non-structural carbohydrate (NSC) content was calculated as the sum of St and SS. Soluble protein (SP) was quantified using the Coomassie Brilliant Blue G-250 method (Li, 2000). Total carbon (C) content was analyzed with a TOC analyzer (Analytik Jena AG, Jena, Germany) (Khanina, Georgiyants & Khanin, 2023).

### Determination of petal mineral elements content

Oven-dried petal samples (0.5 g) were digested with a 5:1 (v/v) mixture of HNO_3_ and HClO_4_ until the solution became clear. After filtration and dilution to 50 mL, the concentrations of total phosphorus (P), total potassium (K), total calcium (Ca), total magnesium (Mg), total zinc (Zn), total boron (B), total iron (Fe), total manganese (Mn), total sulfur (S) were determined by inductively coupled plasma mass spectrometry (ICP-MS, Thermo Fisher Scientific Inc, Germany) (Kararenk et al., 2025). Total nitrogen (N) was measured using a continuous flow analyzer (AA3; Bran-Luebbe Inc.) (Zhang et al., 2014).

### Determination of petal morphological structure

Petal anatomical structure was examined using the paraffin sectioning method (He et al., 2024; Li et al., 2024; Li, Yang & Chen, 2022). First, mid-segment petal tissues were fixed in FAA fixative (formaldehyde: glacial acetic acid: 70% ethanol = 5:5:90 by volume) for at least 24 h. The samples were subsequently dehydrated through a graded ethanol series, cleared in xylene, and infiltrated with paraffin at 65 °C before embedding. The embedded tissues were sectioned into approximately 4 μm-thick slices using a microtome. After deparaffinization in xylene and rehydration through a descending ethanol series, the sections were stained with safranin, cleared again in xylene, and finally mounted with neutral balsam. Whole-section images were acquired using a digital slide scanner (P250 FLASH III, 3DHISTECH, Hungary). The morphological characteristics of the upper and lower epidermal cells were directly observed under a light microscope using water-mounted temporary slides. The following morphological parameters were measured using Image J software (National Institutes of Health, USA): petal thickness (PT), upper epidermal thickness (UET), lower epidermal thickness (LET), parenchyma tissue thickness (PTT), upper epidermal cell width (UEW), and lower epidermal cell width (LEW). Additionally, the thickness-to-width ratio (TWR) of the upper epidermal cells was calculated using the formula: tWR = UET/UEW.

### Integrated assessment of petal morphological, nutritional, antioxidant, and mineral traits

A comprehensive evaluation method based on radar chart analysis was applied with minor modifications (Huang et al., 2024). The petal morphological structure index (PMSI) was calculated as:

(1) \begin{eqnarray*}\mathrm{PMSI}=0.5\times {\mathop{\sum \nolimits }\nolimits }_{i}^{n}{\mathrm{SL}}_{i}^{2}\times \mathrm{sin}(\pi /\mathrm{n})\end{eqnarray*}

(2) \begin{eqnarray*}S{L}_{i}=X/{X}_{max}\end{eqnarray*}

(3) \begin{eqnarray*}S{L}_{i}=1-X/{X}_{max}.\end{eqnarray*}

Here, n represents the number of parameters, *SL*_*i*_ denotes the standardized value (0–1), *X* is the measured value, and *X*_*max*_ is the maximum value among samples. For positive indicators (where higher values are preferable), Formula (2) was applied; for negative indicators (where lower values are better; MDA was the only negative indicator in this study), Formula (3) was applied. PT, UET, LET, PTT, and UEW were selected for PMSI. Similarly, the petal nutrient index (PNI) included C, SS, St, NSC, and SP; the petal antioxidant index (PAI) comprised MDA, SOD, POD, CAT, and APX; and the petal mineral element index (PMEI) included N, P, K, Ca, Mg, Zn, B, Fe, Mn, and S.

### Comprehensive membership function evaluation during flower development

A membership function approach (Zhang et al., 2025) was used to standardize trait values between 0 and 1:

(4) \begin{eqnarray*}R({X}_{i})=({X}_{i}-{X}_{min})/({X}_{max}-{X}_{min}).\end{eqnarray*}

Here, *X*_*i*_ is the measured value, *X*_*min*_ and *X*_*max*_ are the minimum and maximum values across all flowering stages, and R(Xi) is the membership degree. The overall performance at each stage was ranked based on the sum of membership values.

### Data processing

All data are presented as mean ± standard deviation. Significant differences were assessed by one-way analysis of variance (ANOVA) followed by Tukey’s HSD test (*P* <0.05) in SPSS 25.0 (SPSS Inc., Chicago, IL, USA). Graphs, including line charts, bar charts, correlation heatmaps, and PCA plots, were generated using Origin 2019b (Origin Lab, Northampton, MA, USA). Statistical analyses were performed with SPSS 25.0.

## Results

### Coloration characteristics of *Rosa* ‘Creamy Eternity’ during flower development

Coloration characteristics during flower development of *Rosa* ‘Creamy Eternity’ are shown in Table 1. Significant differences (*P* < 0.05) were observed in color parameters *L*^∗^, *a*^∗^, *b*^∗^, *C*^∗^, *h*^∗^ among the stages. For *L*^∗^, S3, S4, and S5 showed the highest values (93.71, 92.27, and 92.05, respectively), S1 and S2 showed the lowest values (80.31 and 79.99, respectively). For *a*^∗^, S4 showed the highest value (−4.47), S1 the lowest value (−11.91). For *b*^∗^, S2 showed the highest value (43.15), S4 and S5 showed the lowest values (25.14 and 28.36, respectively). For *C*^∗^, S1, and S2 showed the highest values (44.04 and 43.74, respectively), S3, S4 and S5 showed the lowest values (31.70, 25.53 and 29.00, respectively). For *h*^∗^, S1 showed the highest values (105.70), S2 and S3 showed the lowest values (99.36 and 99.01, respectively). Overall, the color parameters of *Rosa* ‘Creamy Eternity’ changed during flowering, manifested as increased brightness, reduced yellow and green intensity, and decreased saturation.

**Table 1:** Color parameters during flower development. Values are averages ± standard deviation (*n* = 3). The *P*-values for L*, a*, b*, C*, and h* were based on a one-way ANOVA. Different lowercase letters indicate significant differences among cultivars (*P* < 0.05). Lightness, L*; Green-Red Axis, a*; Blue-Yellow Axis, b*; Chroma, C**; Hue, h*. Same as follows.

Stage	*L* ^∗^	*a* ^∗^	*b* ^∗^	*C* ^∗^	*h* ^∗^
S1	80.31 ± 1.24 b	−11.91 ± 1.53 c	42.39 ± 6.24 ab	44.04 ± 6.35 a	105.7 ± 1.25 a
S2	79.99 ± 3.53 b	−7.11 ± 0.84 b	43.15 ± 5.51 a	43.74 ± 5.57 a	99.36 ± 0.09 c
S3	93.71 ± 0.23 a	−4.97 ± 0.49 ab	31.31 ± 2.73 bc	31.7 ± 2.77 b	99.01 ± 0.13 c
S4	92.27 ± 3.29 a	−4.47 ± 0.43 a	25.14 ± 0.89 c	25.53 ± 0.85 b	100.09 ± 1.16 bc
S5	92.05 ± 0.28 a	−6.04 ± 0.64 ab	28.36 ± 4.25 c	29 ± 4.29 b	101.7 ± 0.7 b

**Figure 2:**
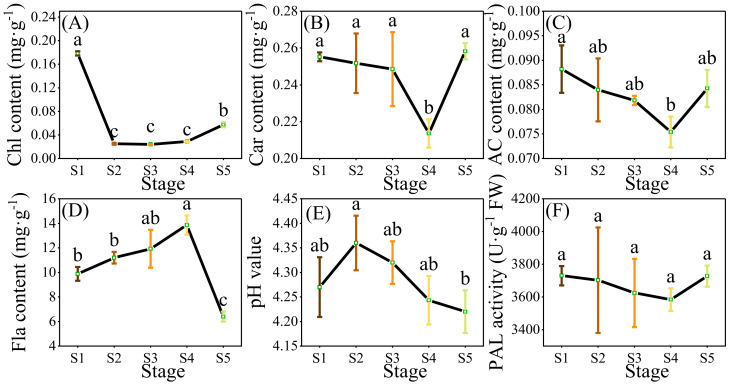
Pigment content, pH, and phenylalanine ammonialyase (PAL) activity during flower development. Values are averages ± standard deviation (*n* = 3). The *P*-values for Chl, Car, AC, Fla, pH and PAL were based on a one-way ANOVA. Different lowercase letters indicate significant differences among stages (*P* < 0.05). Chlorophyll content, Chl; Carotenoid content, Car; Anthocyanin content, AC; Flavonoid content, Fla; Potential of hydrogen, pH; Phenylalanine ammonialyase activity, PAL. Same as follows.

To explore the physiological basis of this color change, we measured pigment content, pH and PAL activity, as pigment content directly determines petal coloration, pH influences anthocyanin stability and color hue, and PAL is a key enzyme in the flavonoid biosynthesis pathway. Pigment content, pH, and PAL activity during flower development of *Rosa* ‘Creamy Eternity’ are shown in Fig. 2. Significant differences (*P* < 0.05) were observed in Chl, Car, AC, Fla, and pH among the stages, while no significant difference (*P* > 0.05) was observed in PAL. For Chl, S1 showed the highest value (0.18 mg g^−1^), S2, S3 and S4 showed the lowest values (0.03, 0.02 and 0.03 mg g^−1^, respectively). For Car, S1, S2, S3 and S5 showed the highest values (0.26, 0.25, 0.25 and 0.26 mg g^−1^, respectively), S4 showed the lowest value (0.21 mg g^−1^). For AC, S1 showed the highest value (0.88 mg g^−1^), S4 showed the lowest value (0.75 mg g^−1^). For Fla, S4 showed the highest value (13.86 μmol g^−1^), S5 showed the lowest value (6.40 μmol g^−1^). For pH, S2 showed the highest value (4.36), S5 showed the lowest value (4.22). Overall, the pigment content of *Rosa* ‘Creamy Eternity’ changed significantly during flowering, governing the presentation of its petal color.

**Figure 3:**
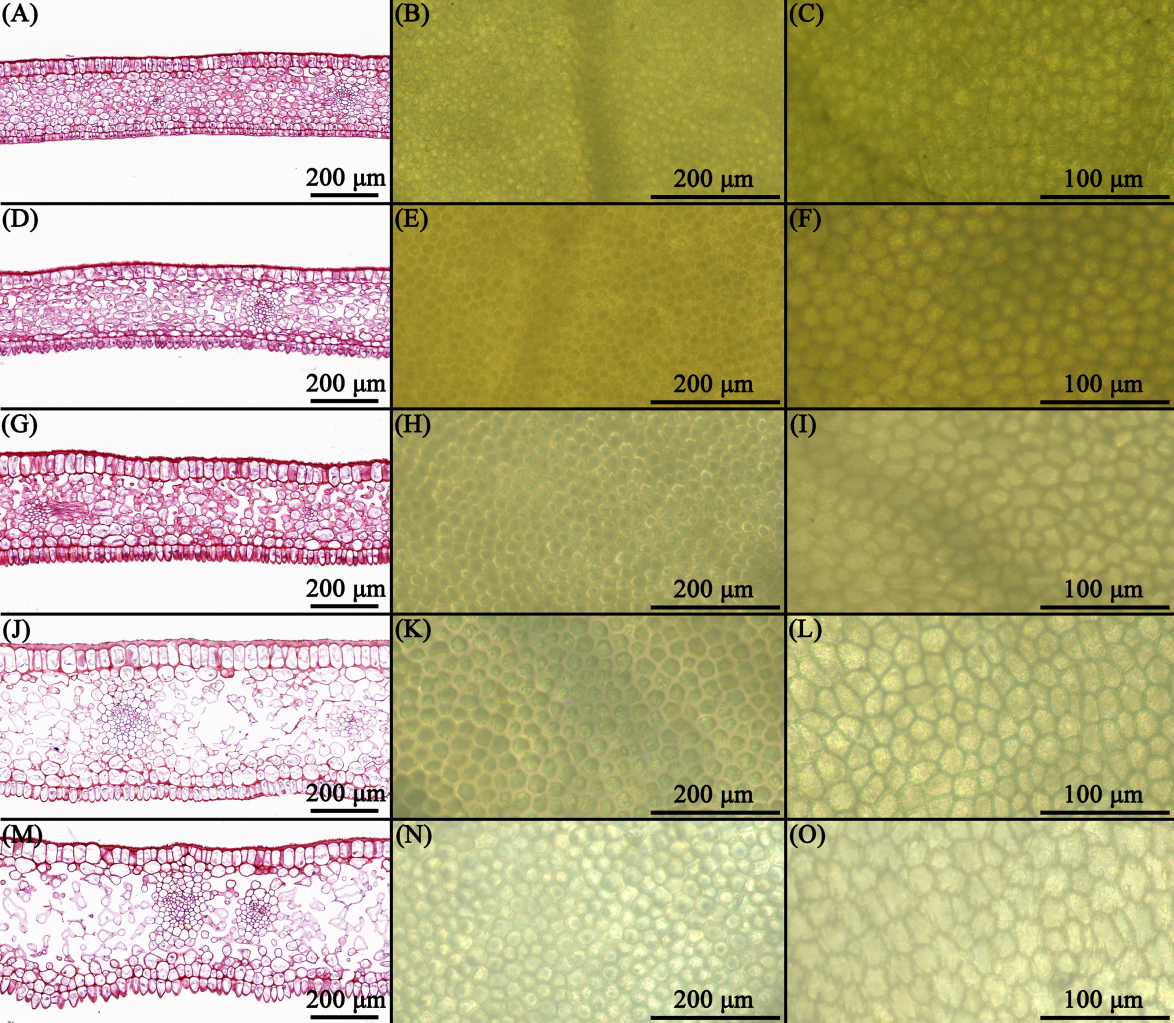
Morphological structure during flower development. A portion of the petal samples were transversely sectioned and paraffin-embedded, with micrographs captured at 10× magnification to observe the sectional structure; another portion was made into temporary water-mounted slides, and micrographs were captured at 20× magnification for microscopic examination of both the abaxial and adaxial epidermal cells. S1 (A, B, C), S2 (D, E, F), S3 (G, H, I), S4 (J, K, L), S5 (M, N, O). Cross-sectional views (A, D, G, J, M ; scale bar: 200 µm), adaxial epidermis (B, E, H, K, N; scale bar: 200 µm), and abaxial epidermis (C, F, I, L, O ; scale bar: 100 µm) are shown.

### Morphological structure indicators of *Rosa* ‘Creamy Eternity’ during flower development

As petals gradually open, the morphological structures of petal cells, including PT, UET, LET, PTT, UEW, LEW, and TWR, undergo marked changes alongside color variation throughout flowering. These structural parameters serve as important indicators of petal development and also influence petal coloration. Morphological structure during flower development were shown in Fig. 3. Morphological structure indicators during flower development of *Rosa* ‘Creamy Eternity’ are shown in Table 2. Significant differences (*P* < 0.05) were observed in PT, UET, LET, PTT, UEW, LEW and TWR among the stages. For PT, S4 and S5 showed the highest values (387.60 and 401.40 µm, respectively), S1 and S2 showed the lowest values (180.47 and 209.60 µm, respectively). For UET, S5 showed the highest value (49.73 μm), S1, S2 and S2 showed the lowest values (38.07, 40.97 and 41.4 µm, respectively). For LET, S5 showed the highest value (44.30 μm), S1 showed the lowest value (17.30 μm). For PPT, S4 and S5 showed the highest values (301.27 and 307.37 µm, respectively), S1 and S2 showed the lowest values (125.10 and 142.67 µm, respectively). For UEW, S5 showed the highest value (35.17 μm), S1 showed the lowest value (12.50 μm). For LEW, S5 showed the highest value (20.00 μm), S1 showed the lowest value (9.53 μm). For TWR, S1 showed the highest value (3.06), S3, S4 and S5 showed the lowest values (1.61, 1.52 and 1.42 µm, respectively). Overall, as the flowers of *Rosa* ‘Creamy Eternity’ progressed to anthesis, the cellular and tissue structures of the petals continuously elongated and expanded, attaining their maximum dimensions by the full bloom stage.

**Table 2:** Morphological structure during flower development. Values are averages ± standard deviation (*n* = 3). The *P*-values for PT, UET, LET, PTT, UEW, LEW and TWR were based on a one-way ANOVA. Different lowercase letters indicate significant differences among stages (*P* < 0.05). Petal thickness, PT; Upper epidermal thickness, UET; Lower epidermal thickness, LET; Parenchyma tissue thickness, PTT; Upper epidermal cell width, UEW; Lower epidermal cell width, LEW; Petal thickness and width ratio, TWR. Same as follows.

Stage	PT	UET	LET	PTT	UEW	LEW	TWR
	µm	µm	µm	µm	µm	µm	/
S1	180.47 ± 14.71 c	38.07 ± 2.2 b	17.3 ± 0.95 d	125.1 ± 13.39 c	12.5 ± 0.75 d	9.53 ± 0.91 d	3.06 ± 0.29 a
S2	209.6 ± 4.4 c	40.97 ± 3.37 b	25.97 ± 1.44 c	142.67 ± 6.55 c	18.9 ± 0.78 c	11.83 ± 0.6 cd	2.17 ± 0.17 b
S3	270.03 ± 15.27 b	41.4 ± 1.15 b	38.37 ± 2.51 b	182.27 ± 12.85 b	25.63 ± 0.42 b	13.83 ± 0.4 c	1.61 ± 0.02 c
S4	387.6 ± 9.13 a	43.73 ± 4.22 ab	42.6 ± 1.8 ab	301.27 ± 7.82 a	28.87 ± 1.46 b	16.93 ± 0.87 b	1.52 ± 0.22 c
S5	401.4 ± 6.5 a	49.73 ± 3.07 a	44.3 ± 2.55 a	307.37 ± 6.21 a	35.17 ± 3.31 a	20 ± 1.4 a	1.42 ± 0.17 c

### Nutrient content of *Rosa* ‘Creamy Eternity’ during flower development

**Figure 4:**
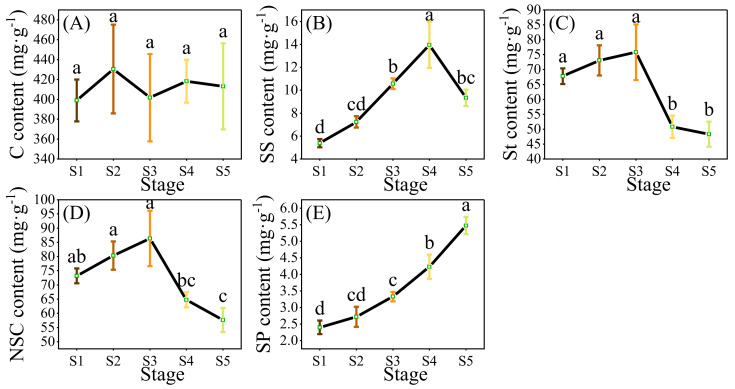
Nutrient content during flower development. Values are averages ± standard deviation (*n* = 3). The *P*-values for C, SS, St, NSC and SP were based on a one-way ANOVA. Different lowercase letters indicate significant differences among stages (*P* < 0.05). Total carbon, C; Soluble sugars, SS; Starch, St; Non-structural carbohydrates, NSC; Soluble protein, SP. Same as follows.

C serves as the main structural component of petals, while NSC, including SS, St, and SP, provide essential material and energy support throughout the flowering process. Nutrient content during flower development of *Rosa* ‘Creamy Eternity’ are shown in Fig. 4. Significant differences (*P* < 0.05) were observed in SS, St, NSC and SP among the stages, while no significant difference (*P* > 0.05) was observed in C. For SS, S4 showed the highest value (13.96 mg g^−1^), S1 showed the lowest value (5.38 mg g^−1^). For St, S1, S2 and S3 showed the highest values (67.80, 73.11 and 75.81 mg g^−1^, respectively), S4 and S5 showed the lowest values (50.81 and 48.31 mg g^−1^, respectively). For NSC, S2 and S3 showed the highest values (80.34 and 86.37 mg g^−1^, respectively), S5 showed the lowest value (57.65 mg g^−1^). For SP, S5 showed the highest value (5.47 mg g^−1^), S1 showed the lowest value (2.40 mg g^−1^). Overall, the nutrient content of *Rosa* ‘Creamy Eternity’ changed significantly during flowering, suggesting that the synthesis and consumption of nutrients facilitated flower opening.

### Antioxidant capacity of *Rosa* ‘Creamy Eternity’ during flower development

**Figure 5:**
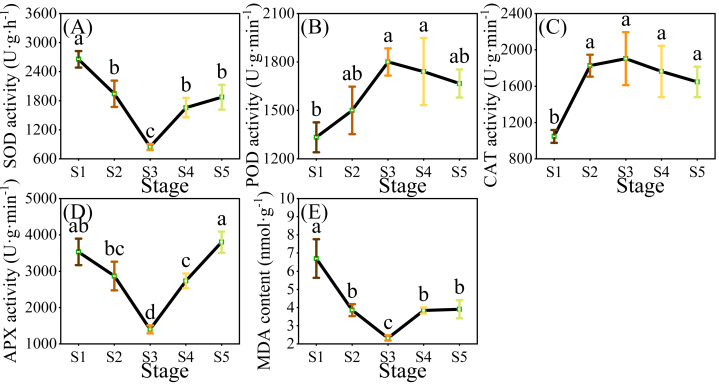
Antioxidant capacity during flower development. Values are averages ± standard deviation (*n* = 3). The *P*-values for SOD, POD, CAT, APX and MDA were based on a one-way ANOVA. Different lowercase letters indicate significant differences among stages (*P* < 0.05). Superoxide dismutase activity, SOD; Peroxidase, POD; Catalase, CAT; Ascorbic acid peroxidase activity, APX; Malondialdehyde, MDA. Same as follows.

Stress tolerance is also critical for successful flowering. The activities of antioxidant enzymes such as SOD, POD, CAT, and APX determine the plant’s ability to withstand environmental stress during flowering. MDA content serves as a key indicator of oxidative damage, with higher values reflecting more severe oxidative stress during flowering. Antioxidant capacity during flower development of *Rosa* ‘Creamy Eternity’ are shown in Fig. 5. Significant differences (*P* < 0.05) were observed in SOD, POD, CAT, APX and MDA among the stages. For SOD, S1 showed the highest value (2,655.01 U g h^−1^), S3 showed the lowest value (845.53 U g h^−1^). For POD, S3 and S4 showed the highest values (1,800.00 and 1,740.00 U g h^−1^, respectively), S1 showed the lowest value (1,333.33 U g h^−1^). For CAT, S2, S3, S4 and S5 showed the highest values (1,826.62, 1,902.60, 1,762.25 and 1,647.30 U g h^−1^, respectively), S1 showed the lowest value (1,046.85 U g h^−1^). For APX, S5 showed the highest value (3,533.34 U g h^−1^), S3 showed the lowest value (1,399.99 U g h^−1^). For MDA, S1 showed the highest value (6.70 nmol⋅g^−1^), S3 showed the lowest value (2.32 nmol⋅g^−1^). Overall, the antioxidant capacity of *Rosa* ‘Creamy Eternity’ changed significantly during flowering, contributing to the enhancement of antioxidant activity during flower development and regulating MDA content.

### Mineral element content of *Rosa* ‘Creamy Eternity’ during flower development

**Figure 6:**
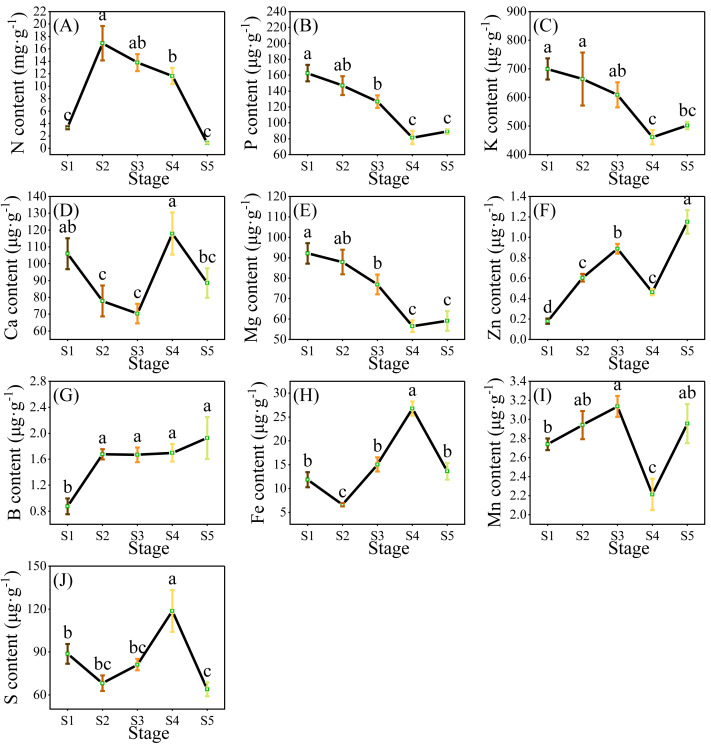
Mineral element content during flower development. Values are averages ± standard deviation (*n* = 3). The *P*-values for C, SS, St, NSC and SP were based on a one-way ANOVA. Different lowercase letters indicate significant differences among stages (*P* < 0.05). Nitrogen content, N; Phosphorus content, P; Potassium content, K; Calcium content, Ca; Magnesium content, Mg; Zinc content, Zn; Boron content, B; Iron content, Fe; Manganese content, Mn; Sulfur content, S. Same as follows.

Mineral elements (N, P, K, Ca, Mg, Zn, B, Fe, Mn, and S) provide the necessary foundation for floral organ development and physiological metabolism. Mineral element content during flower development of *Rosa* ‘Creamy Eternity’ are shown in Fig. 6. Significant differences (*P* < 0.05) were observed in N, P, K, Ca, Mg, Zn, B, Fe Mn and S among the stages. For N, S2 showed the highest value (16.90 mg g^−1^), S1 and S5 showed the lowest values (3.33 and 0.88 mg g^−1^, respectively). For P, S1 showed the highest value (162.52 μg g^−1^), S4 and S5 showed the lowest values (81.49 and 89.10 μg g^−1^, respectively). For K, S1 and S2 showed the highest values (699.34 and 663.92 μg g^−1^, respectively), S4 showed the lowest value (460.59 μg g^−1^). For Ca, S4 showed the highest value (117.95 μg g^−1^), S2 and S3 showed the lowest values (77.85 and 70.28 μg g^−1^, respectively). For Mg, S1 showed the highest value (92.10 μg g^−1^), S4 and S5 showed the lowest values (56.44 and 59.07 μg g^−1^, respectively). For Zn, S5 showed the highest value (1.15 μg g^−1^), S1 showed the lowest value (0.18 μg g^−1^). For B, S2, S3, S4 and S5 showed the highest values (1.68, 1.67, 1.70 and 1.93 μg g^−1^, respectively), S1 showed the lowest value (0.88 μg g^−1^). For Fe, S4 showed the highest value (26.77 μg g^−1^), S2 showed the lowest value (6.58 μg g^−1^). For Mn, S3 showed the highest value (3.14 μg g^−1^), S4 showed the lowest value (2.21 μg g^−1^). For S, S4 showed the highest value (118.65 μg g^−1^), S2 showed the lowest value (63.82 μg g^−1^). Overall, the mineral element content in *Rosa* ‘Creamy Eternity’ changed significantly during flowering, suggesting that dynamic changes in mineral elements occur throughout the flower opening process.

**Figure 7:**
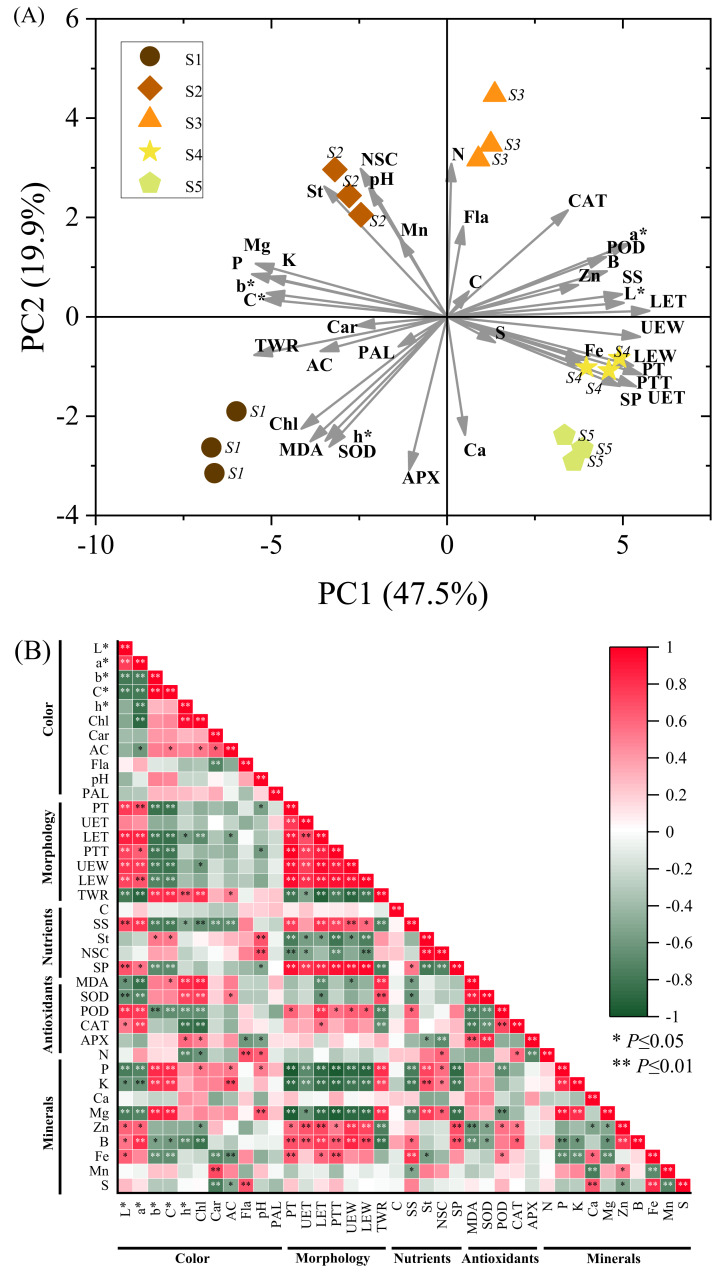
Principal Component Analysis (PCA) (A) and correlation (B) analysis of color, morphology, nutrients, antioxidants and minerals during flower development. PCA was employed to compare the comprehensive changes in indicators across developmental stages. Principal Component 1 (PC1) and Principal Component 2 (PC2) accounted for the first and second highest contribution rates, respectively. Gray arrows represent the measured variables. Arrows pointing in similar directions with acute angles indicate positive correlations between corresponding variables, whereas obtuse angles denote negative correlations. Colored dots correspond to distinct flowering stages; closer proximity of a dot to an arrow indicates a higher value for that specific variable in the respective stage. The correlations in the figure were calculated using Pearson (*n* = 15, 3 biological replicates × 5 stages). * indicates *P* ≤ 0.05, and ** indicates *P* ≤ 0.01. Red indicates a positive correlation, while green indicates a negative correlation; the darker the color, the higher the correlation.

### Relationships among various indicators of *Rosa* ‘Creamy Eternity’ during flower development

Principal component analysis (PCA, Fig. 7A) and correlation heatmaps (Fig. 7B) collectively revealed associations between different flowering stages and various indicators, as well as intrinsic correlations among the indicators themselves. The category-wise PCA and correlation analysis results are provided in Supplemental Information 2. Principal Components 1 and 2 together contributed 67.4% of the explained variance (Fig. 7A). For color parameters and pigments, AC, Chl, Car and *h*^∗^ were closely associated with S1, indicating that these metrics were highest at the bud stage. As flowering progressed, *L*^∗^ increased, reflecting a shift toward lighter and brighter petals. Chl and Car showed positive correlations with AC, while Fla were positively correlated with Car. For morphological structure, UET, LEW, PTT, PT, UEW and LET were predominantly associated with S4 and S5, indicating that these structural traits peaked in the late flowering stages. In contrast, TWR was higher in S1 and S2 and decreased as flowering progressed. All the aforementioned morphological parameters showed positive correlations with each other, whereas TWR was negatively correlated with all of them. For nutrient content, NSC and St were mainly concentrated in S1 to S3, indicating higher levels in early flowering stages. In contrast, SS and SP were highest in S4 and S5. NSC was positively correlated with starch, while SP showed a positive correlation with SS but negative correlations with starch and NSC. For antioxidant indices, MDA was highest in S1, suggesting significant osmotic stress at the bud stage. SOD, POD, CAT and APX were most active in S4 and S5, indicating stronger antioxidant capacity during initial and full bloom. MDA was positively correlated with SOD and APX, but negatively correlated with POD and CAT. SOD also showed a negative correlation with both POD and CAT. For mineral elements, N, P, K, Mg, and Mn were higher in S1 to S3, whereas Ca, Fe, and S were highest in S4, and Zn and B peaked in S5, reflecting varying mineral requirements across flowering stages. P was positively correlated with K and Mg, but all three showed negative correlations with B and Fe. Ca was positively correlated with Fe and S, and negatively correlated with Zn and Mn.

From a stage-specific perspective, S1 showed strong associations with pigments (Chl, AC, and Car), the highest PAL activity, and elevated TWR, SOD, and MDA levels. S2 was marked by higher pH and increased NSC and St content. S3 featured higher N and Fla content. S4 and S5, meanwhile, exhibited peak morphological traits, maximum lightness, and the highest levels of SS, SP, Zn, B, and Fe.

Significant correlations were observed among different types of indicators (Fig. 7B). Regarding color and nutrients, both SS and SP exhibited significant positive correlations with *L*^∗^ and a. In addition, SS showed significant negative correlations with *b*^∗^, *C*^∗^, *h*^∗^, as well as with Chl, Car, and AC. For antioxidants and minerals, Zn and B were significantly negatively correlated with MDA and SOD, and also showed significant negative correlations with CAT. Regarding nutrients and minerals *versus* morphological traits, most morphological indicators (except TWR) exhibited significant negative correlations with St, P, K, and Mg, while showing significant positive correlations with SP, Zn, and B. Notably, TWR displayed opposite correlation patterns.

In summary, widespread significant correlations exist among different types of indicators, suggesting that floral development is closely associated with morphological, nutritional, antioxidant, and mineral factors, which exhibit a high degree of synergistic coordination throughout the flowering process.

### Comprehensive membership function evaluation of petal morphology and physio-biochemical parameters in *Rosa* ‘Creamy Eternity’ during flower development

**Figure 8:**
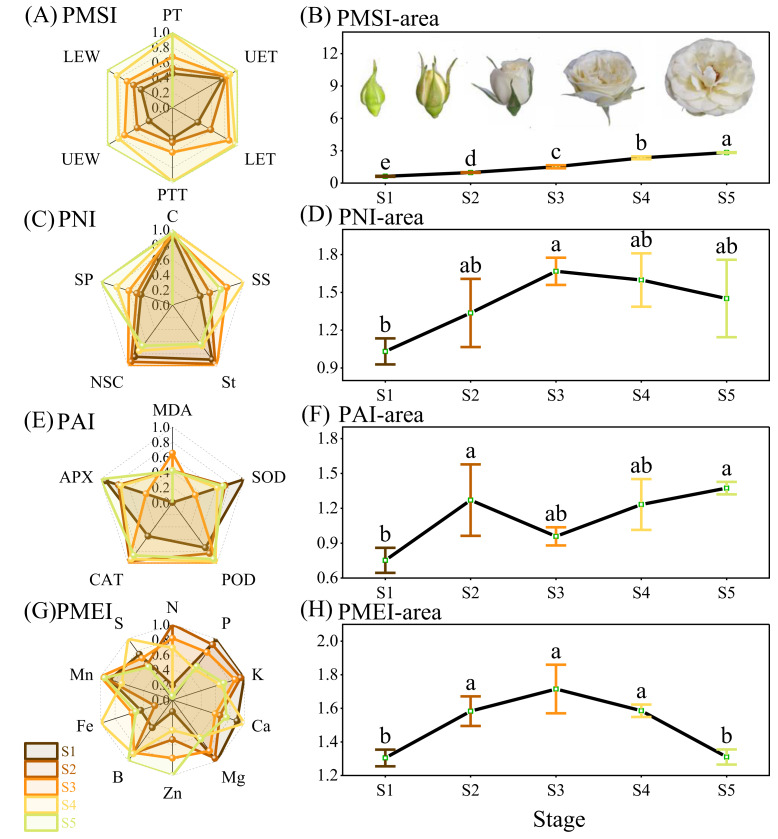
Radar plot of relative petal morphological structure and physio-biochemical parameters (A, C, E, G) combined with petal morphological structure index (PMSI) (B), petal nutrition index (PNI) (D), petal antioxidant index (PAI) (F), and petal. Values are averages ± standard deviation (*n* = 3). The *P*-values for PMSI, PNI, PAI and PMEI were based on a one-way ANOVA. Different lowercase letters indicate significant differences among stages (*P* < 0.05).

As shown in Fig. 8, PMSI, PNI, PAI, and PMEI all showed significant differences (*P* < 0.05) during flower development in *Rosa* ‘Creamy Eternity’. The PMSI increased gradually throughout the flowering process, reaching its highest value at S5 (2.83) and the lowest at S1 (0.63). The PNI increased initially and then stabilized, peaking at S3 (1.67) and being lowest at S1 (1.03). The PAI also increased first and then remained relatively stable, with the highest values at S2 and S5 (1.27 and 1.37, respectively), and the lowest at S1 (0.75). The PMEI increased initially and then decreased, with the highest values observed at S2, S3, and S4 (1.58, 1.72, and 1.59, respectively), and the lowest at S1 and S5 (1.30 and 1.31, respectively).

**Figure 9:**
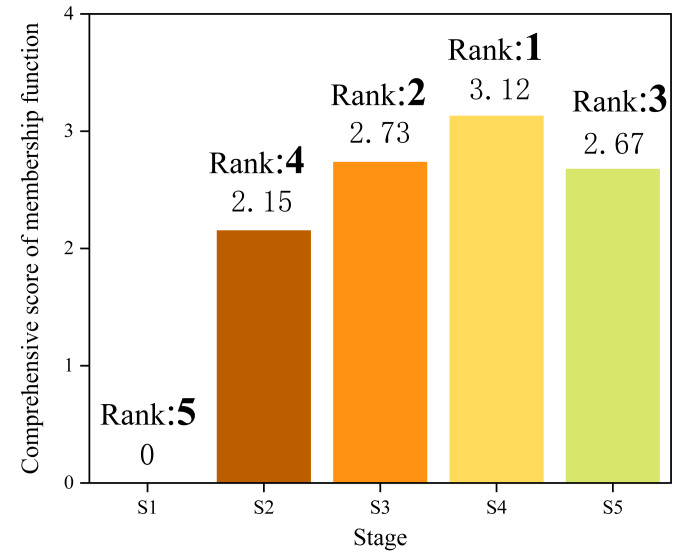
Comprehensive scores and rankings of membership function values for PMSI, PNI, PAI, and PMEI during flower development. A higher comprehensive score indicates a better rank, reflecting superior overall performance at that particular stage.

As shown in Fig. 9, membership degrees for PMSI, PNI, PAI, and PMEI were calculated to derive a comprehensive membership score, where higher values indicate superior overall performance at that particular stage. The cultivars ranked as follows by composite score: S4 (3.12, Rank 1) > S3 (2.73, Rank 2 > S5 (2.67, Rank 3 > S2 (2.15, Rank 4) > S1 (0, Rank 5). Collectively, S4 demonstrated consistently high performance across all flowering stages.

## Discussion

### Changes in pigment content and morphological structure drive petal hue shifts during flower development of *Rosa* ‘Creamy Eternity’

The dynamic changes in flower color during blooming represent one of the most critical ornamental traits in plants. These variations arise not only from alterations in the composition and concentration of petal pigments but may also reflect the plant’s active adaptation to environmental conditions (Enriquez et al., 2023). In *Rosa floribunda*, higher *b*^∗^ values impart a yellowish tone to the petals (Mallick et al., 2024). Similarly, PCA in this study effectively captured shifts in pigment concentrations during flowering. *Rosa* ‘Creamy Eternity’ exhibited a yellow-green hue at early stages, characterized by elevated AC, Car, Chl, and *b*^∗^ values. As flowering progressed, Chl content declined substantially to low levels, while Car and AC also decreased. In contrast, Fla content increased at stage S3, likely contributing to color maintenance. By the full-bloom stage (S5), all four pigments had diminished considerably. Visually, the yellow-green tone gradually faded, reflected by an increase in *a*^∗^ and *L*^∗^ values and a decrease in *b*^∗^ value, resulting in an overall shift from yellow-green to light yellow. This color transition pattern resembles that observed in the yellow-white *Herbaceous peony* cultivar ‘Jinzanciyu’ during its flowering process (Zhang et al., 2023b). Yeon & Kim (2020) analyzed pigment profiles in eight rose cultivars and reported similar findings. Specifically, the white-flowered cultivars *Rosa* ‘Beast’ and ‘Marilyn Monroe’ exhibited extremely low carotenoid levels. In contrast, anthocyanin content was significantly higher in the pink- and red-flowered cultivars, namely ‘Ahoi’ and ‘Pink Condor’ (pink), as well as ‘Pearl Red’ and ‘Vital’ (red). Cheng et al. (2024) similarly found that pink and black-red petals are rich in anthocyanins, with concentrations significantly higher than those in white and yellow petals. In contrast, in *Rosa damascena*, anthocyanin accumulation peaks at full bloom, while flavonol content remains relatively stable, supporting petal color stability. This pattern is primarily attributed to the upregulation of key pigment-related genes such as *CHS* and *CHI* during flowering (Gudarzi et al., 2024). The opposite trend observed in *Rosa* ‘Creamy Eternity’ may result from differential regulatory mechanisms of color-related genes during flower development.

Petal pigment content regulates the degree of light absorption; higher concentrations enhance absorption and deepen flower color (Noda et al., 1994). Darker-colored flowers absorb more solar radiation, raising floral temperature and potentially aiding reproductive development. Under high-temperature conditions, however, brighter petals reflect more light, mitigating radiation damage (Hopkins & Forrest, 2020). Some studies suggest that pigments accumulated under sunlight may provide indirect photoprotection, reducing the adverse effects of intense light on flowering (Koski, Leonard & Tharayil, 2024). Furthermore, flower color may influence reproductive success by affecting pollinator behavior (Enriquez et al., 2023).

The reflective properties of the petal surface play a key role in determining floral texture. Conical epidermal cells with a high TWR direct light toward pigment-rich regions at the cell base, enhancing absorption and deepening color perception (Gkika, Argiropoulos & Rhizopoulou, 2015; Kay, Daoud & Stirton, 1981). In contrast, flattened epidermal cells reflect more light, improving petal brightness (Glover & Martin, 1998). Consequently, the TWR of upper epidermal cells often correlates significantly with petal color intensity. Internal petal structure also modulates color tone. Air spaces distributed among the epidermis, palisade, and spongy tissues enhance light scattering; increases in their volume and number elevate petal brightness (Kay, Daoud & Stirton, 1981). In this study, petal development was accompanied by enlarged and more numerous air spaces, increased UEW, and reduced TWR. These structural changes improved light scattering and reflectance while reducing absorption, collectively leading to brighter petals. PCA indicated that TWR, associated with light absorption, peaked at S1, whereas indicators of petal tissue development reached their highest levels at S5. We hypothesize that these characteristics, including enhanced petal brightness, increased air space volume, and reduced pigment content, may strengthen light reflection and minimize light absorption within the petal tissue, thereby helping to maintain stable floral development under full-sun conditions. This interpretation, while speculative at this stage, merits further validation in follow-up studies through physiological or functional investigations.

### Synergistic regulation of flowering in *Rosa* ‘Creamy Eternity’ by nutrients, antioxidant enzymes, and mineral elements

Nutrients play a direct role in the formation and opening of floral organs. Among them, NSC, composed of St and SS sugars, act as triggers for sugar signaling and provide a continuous carbon source for the flowering process (Li et al., 2021). The underlying mechanisms include: the breakdown of St into SS, which partly serve as energy substrates to stabilize flower color structure, maintain pigmentation and longevity, and partly promote flavonoid synthesis, while also participating in osmotic regulation to maintain cell membrane stability and mitigate external damage (Sanjbod et al., 2023; Clearwater et al., 2021; Da Silva et al., 2013; Ren et al., 2023). Studies have shown that higher NSC content in *Tree peony* contributes to improved flowering quality and ornamental value (Shi et al., 2024). Further research has revealed that the accumulation of NSC, as a key intermediary substance, promotes flowering in *Tree peony*. In rice, the remobilization of NSC serves as a physiological mechanism to effectively cope with high-temperature stress (Mahmood et al., 2024). In bamboo, sugar catabolism accelerates during flowering (Li et al., 2023a). In this study, as the flowering process of *Rosa* ‘Creamy Eternity’ progressed, the contents of NSC, St, and SS all showed an initial increase followed by a decrease. PCA results indicated that NSC and St were closer to stages S1–S3, while SS and SP were closer to S4 and S5. This suggests that these nutrients gradually accumulate in the early flowering stages, preparing in advance for the high metabolic demands of the initial and full-bloom stages to cope with the sudden acceleration of flowering. Notably, the sharp decline in St content and the continuous increase in SS content during S4 may reflect the breakdown of St into SS to maintain the stability of the flowering process. Additionally, SP content gradually increased during flowering, peaking at the full-bloom stage. The reasons for this may include: (1) SP, as a key structural material for floral organ construction, is in high demand during flowering (Sano, Kawashima & Iwai, 1984); (2) SP, as a major component of various metabolic enzymes, participates in regulating multiple physiological processes, including floral fragrance release (Yang et al., 2022; Bai et al., 2010); (3) SP, as an important osmotic regulator, helps maintain cell osmotic potential, ensuring water absorption during rapid petal elongation and expansion, thereby maintaining cell turgor pressure and structural integrity, which is crucial for petal unfolding and morphological maintenance (Gao et al., 2023). In *Rosa* ‘Yametsu-Hime’, SP reaches its maximum at the full-bloom stage (Yang et al., 2024), which is consistent with the findings of this study.

Flowering in plants is a highly energy-consuming physiological process accompanied by enhanced respiration and overall metabolism, inevitably generating ROS such as superoxide anions (O_2_^−^) and H_2_O_2_ (Gong et al., 2018). When ROS accumulate excessively, they cause oxidative damage, irreversibly accelerating flower senescence, leading to dull coloration, shortened lifespan, and reduced ornamental quality (Zhang et al., 2006). Environmental stresses such as high temperatures, drought, and strong light during flowering can further exacerbate this process (Shani, Ditta & Khan, 2025; Kourani et al., 2025). Antioxidant enzymes mainly include SOD, CAT, POD, and APX. Among them, SOD first converts superoxide anions into H_2_O_2_ through a disproportionation reaction, after which POD, CAT, and APX jointly catalyze its decomposition into H_2_O and O_2_, thereby reducing ROS accumulation and membrane lipid peroxidation damage, lowering MDA content, and ensuring the stability of the flowering process and flower quality (Yuan et al., 2020). During metabolically active stages such as flower initiation and expansion, the activities of these enzymes typically increase to cope with internal oxidative stress (Cai et al., 2019). Thamaraiselvi et al. (2019) observed that antioxidant enzyme activities increase during petal senescence in *Rosa bourboniana* Desp. and *Rosa centifolia* L., in response to both elevated ethylene production and a decline in respiratory activity. However, Önder (2023) reported contrasting results in oil-bearing rose (*Rosa damascena* Mill.), where the activities of antioxidant enzymes, including SOD, CAT, glutathione reductase (GR), and POD, were highest at the bud stage and lowest in fully opened flowers. These discrepancies may be attributed to species-specific differences in flowering stage characteristics among rose cultivars, which could ultimately determine their intended applications, such as product development or landscape use. In this study, SOD activity initially increased and then decreased, while POD and CAT showed the opposite trend. Correlation analysis revealed a highly significant negative correlation between SOD and MDA with POD and CAT, indicating that the synergistic action of these three enzymes efficiently clears ROS, ultimately maintaining MDA at a low level. APX, which specifically scavenges ROS in chloroplasts, exhibited a trend similar to SOD activity. However, Pearson correlation coefficients reflect only the strength and direction of linear relationships and do not imply direct comparability between indicator values; thus, they serve as auxiliary references for understanding synergistic patterns. PCA results showed that MDA accumulated more in the S1 stage, while SOD, CAT, POD, and APX were closer to the S4 and S5 stages. This suggests that MDA accumulation in the early flowering stage may activate the antioxidant enzyme system, thereby alleviating oxidative damage during the full-bloom stage and ensuring flowering quality. In summary, antioxidant enzymes work synergistically to reduce oxidative damage in petals and maintain the stability of the flowering process.

Mineral elements not only provide energy and structural components for plants but also act as regulatory molecules involved in growth and development (Sanagi, Rolland & Sato, 2025). Macronutrients N, P, and K, as fundamental components, participate in floral organ construction and regulate flowering time (Jun, Shim & Park, 2023; Ye et al., 2019). N is a key component of proteins, nucleic acids, and enzymes, and its content directly determines the plant’s nutrient accumulation capacity (Yu et al., 2023). The development of floral organs such as petals, sepals, stamens, and pistils relies on nitrogen support (Yu et al., 2023). P, as a component of ATP, is a core carrier for energy transfer during flowering. It regulates the absorption and distribution of phosphorus through transporters such as PHPT1 and PHO1, ensuring energy supply to floral organs (Jones & Jones, 2009; Dai et al., 2024). K, by regulating cell elongation, maintaining osmotic balance, and enhancing stress resistance, ensures petal morphogenesis and flowering stability (Beauzamy, Nakayama & Boudaoud, 2014; Meena & Prajapati, 2022). In *Theobroma cacao*, low levels of N, P, and K significantly delay flowering, reduce flower number, and decrease flowering intensity (Weinstein et al., 2024). In *Gladiolus grandiflorus*, appropriate supplementation of N, P, and K improves flowering quality and extends the flowering period (Singh et al., 2024). In this study, the contents of N, P, and K in *Rosa* ‘Creamy Eternity’ were higher in the early flowering stages but generally showed a declining trend thereafter (except for N, which increased during S1–S2). Similarly, Önder (2023) reported comparable findings in oil-bearing rose (*Rosa damascena* Mill.), where mineral element content exhibited a declining trend as flowering progressed. This may be due to increased nutrient demands during flowering or a dilution effect caused by rising biomass. Additionally, secondary macronutrients such as Ca, Mg, and S, as well as micronutrients like B, Zn, and Fe, play important roles in enzymatic reactions and hormone synthesis, collectively participating in the regulation of flower development and reproduction (Jun, Shim & Park, 2023). The dynamic changes in mineral elements during flowering are regulated by the plant’s nutritional needs. For example, in walnut, the contents of P, Zn, and Cu are higher in the early flowering stage, while K, Fe, Mn, Ca, and Mg increase during the flowering stage (Hoque, Achanai & Nattawut, 2014). This study revealed that mineral element contents generally exhibited fluctuating changes. Further PCA and correlation analysis indicated that *Rosa* ‘Creamy Eternity’ has varying demands for different mineral elements at different flowering stages. These elements exhibit complex correlations and work together to coordinately regulate the nutrient supply during flowering.

### Comprehensive analysis of *Rosa* ‘Creamy Eternity’ during flower development

Roses possess not only high ornamental value in landscaping but also demonstrate significant potential for application and development (Shariatzadeh, Bijani & Bahadori, 2025). Radar chart analysis revealed that as flowering progressed, the petal tissue structure gradually improved, reaching its optimal developmental state at the full-bloom stage (Figs. 8A, 8B). Concurrently, nutrient levels showed progressive accumulation (Figs. 8C, 8D), likely because flowering, as a highly energy-consuming physiological process, requires both carbohydrates for energy provision and proteins for structural construction. The coordinated accumulation of these components provides the material foundation for normal floral organ development (Borghi, 2025). Furthermore, antioxidant enzyme activities generally exhibited an upward trend (Figs. 8E, 8F), which may be related to the gradual accumulation of oxidative stress during flower opening and reflects the increased environmental pressures faced by fully expanded flowers at the full-bloom stage, leading to a corresponding enhancement in antioxidant capacity. Some studies have reported that *Rosa* ‘Carola’ and *Rosa* ‘Iceberg’ show the highest 2,2-diphenyl-1-picrylhydrazyl (DPPH) radical scavenging capacity and antioxidant activity at the initial flowering stage (Hou, Pan & Zhang, 2014), which differs from our findings. This discrepancy may be attributed to variations in inherent antioxidant capacity among cultivars and influences of cultivation conditions.

Mineral nutrients directly affect flowering time, floral quality, and reproductive success: adequate nutrients promote floral development, whereas deficiency impairs it (Jia et al., 2016; Zhang et al., 2023a). In this study, the measured mineral element contents fell within the normal range compared with previous research and did not reach levels of excessive stress (Önder, 2023; Yang et al., 2025). As long as the content of each element remains below its toxicity threshold, a higher concentration is more favorable for flowering. Consequently, for the mineral element contents in this study, higher values are considered better. Regarding mineral elements, their contents generally displayed an initial increase followed by a decrease (Figs. 8G, 8H). This pattern can be explained by the continuous translocation of mineral elements into petals during early flowering due to rapid cell and tissue development (Clarkson & Hanson, 1980), followed by a decline caused by a dilution effect as petal biomass continuously increased (Verlinden, 2003). However, in terms of total content, the absolute amounts of mineral elements should still show a gradual increasing trend as petals develop toward full bloom. From the perspective of overall floral organ development, the comprehensive membership function scores progressively increased from S1 to S4, indicating continuous improvement in floral organs and ongoing accumulation of nutrients and stress resistance in preparation for full bloom. Similarly, in *Hemerocallis citrina*, the highest levels of various compounds at the initial flowering stage, which decline at full bloom, may reflect a strategy of accumulating reserves early to support the flowering process (Mu et al., 2025); our results are consistent with this pattern. Other studies have found that *Hypericum perforatum* contains higher levels of various bioactive components at full bloom (Piatti et al., 2023), while *Artemisia abrotanum* peaks in total protein and free amino acid content at this stage (Cansever & Söğüt, 2022). Mallick et al. (2024) compared the color, composition, bioactive compounds, and antinutrient profiles of ten rose cultivars. They identified *Rosa polyantha*, Hybrid tea rose, and *Rosa grandiflora* as having the highest vitamin E, phenolic, and flavonoid contents, while *Rosa meidrifora* showed the strongest radical scavenging activity. Cluster analysis further suggested that *Rosa* ’Fragrant Delight’, *Rosa floribunda*, *Rosa meidrifora*, *Rosa damascena*, Hybrid tea rose, and *Rosa* Tradescant could serve as valuable sources of bioactive compounds for cosmetics, food colorants, and pharmaceuticals. In contrast to their focus on functional compound screening, our study emphasizes landscape application by evaluating the overall performance of a single cultivar across flowering stages. By integrating multiple indicators, we identified the optimal stage for ornamental value and growth performance, offering practical insights for cultivation and landscape management. In our study, *Rosa* ‘Creamy Eternity’ achieved the highest score at stage S4, indicating optimal overall performance and identifying this as a critical period for floral organ development. Therefore, subsequent cultivation management should emphasize fertilization regulation during early flowering stages to ensure ideal landscape effects at full bloom.

## Conclusion

Significant differences were observed in petal color, pigment content, morphological structure, nutrient content, antioxidant enzyme activity, and mineral element content during flower development (from the flower bud stage to full bloom) of *Rosa* ‘Creamy Eternity’. As flowering progressed, petal color shifted from yellow-green to light yellow with increasing brightness. Structurally, cell expansion and tissue development progressively refined petal morphology, with increased air space volume and a transition from conical to flattened epidermal cells enhancing light reflectance. In nutrient metabolism, NSC accumulated during early flowering, followed by St decomposition into SS to maintain energy supply, while SP content continued to rise. The antioxidant enzyme system acted synergistically to alleviate oxidative damage, peaking in activity at full bloom. Mineral elements interacted in complex ways to coordinately regulate nutrient allocation during flowering, with varying elemental requirements across stages. Overall, *Rosa* ‘Creamy Eternity’ ensures successful full bloom through nutrient accumulation, enhanced antioxidant capacity, and coordinated mineral element supply. Stage S4 is critical for floral organ development, and we recommend strengthened fertilization management during early flowering to optimize landscape performance at full bloom.

## Supplemental Information

10.7717/peerj.21329/supp-1 Supplemental Information 1 Environmental records.

10.7717/peerj.21329/supp-2 Supplemental Information 2 PCA and correlation analysis of color parameters and pigments (A, B), morphological structure (C, D), nutrients (E, F), antioxidant activity (G, H), and mineral elements (I, J) during flower development

10.7717/peerj.21329/supp-3 Supplemental Information 3 As flowers bloom, the color parameters, morphological structure, and physiological and biochemical indicators exhibit fluctuating/variable changes

## Additional Information and Declarations

### Data Availability

The following information was supplied regarding data availability: The raw measurements are available in the Supplementary Files.
