# Supplementary material for: Dynamics of petal pigments and morpho-physiological traits during flower development in Rosa ‘Creamy Eternity’
Source: PeerJ. 2026 Aug 6;14:e21329. doi: 10.7717/peerj.21329 (PMC13453145; doi:10.7717/peerj.21329)
Supplement: Supplementary file 1 [file peerj-14-21329-s002.pdf]

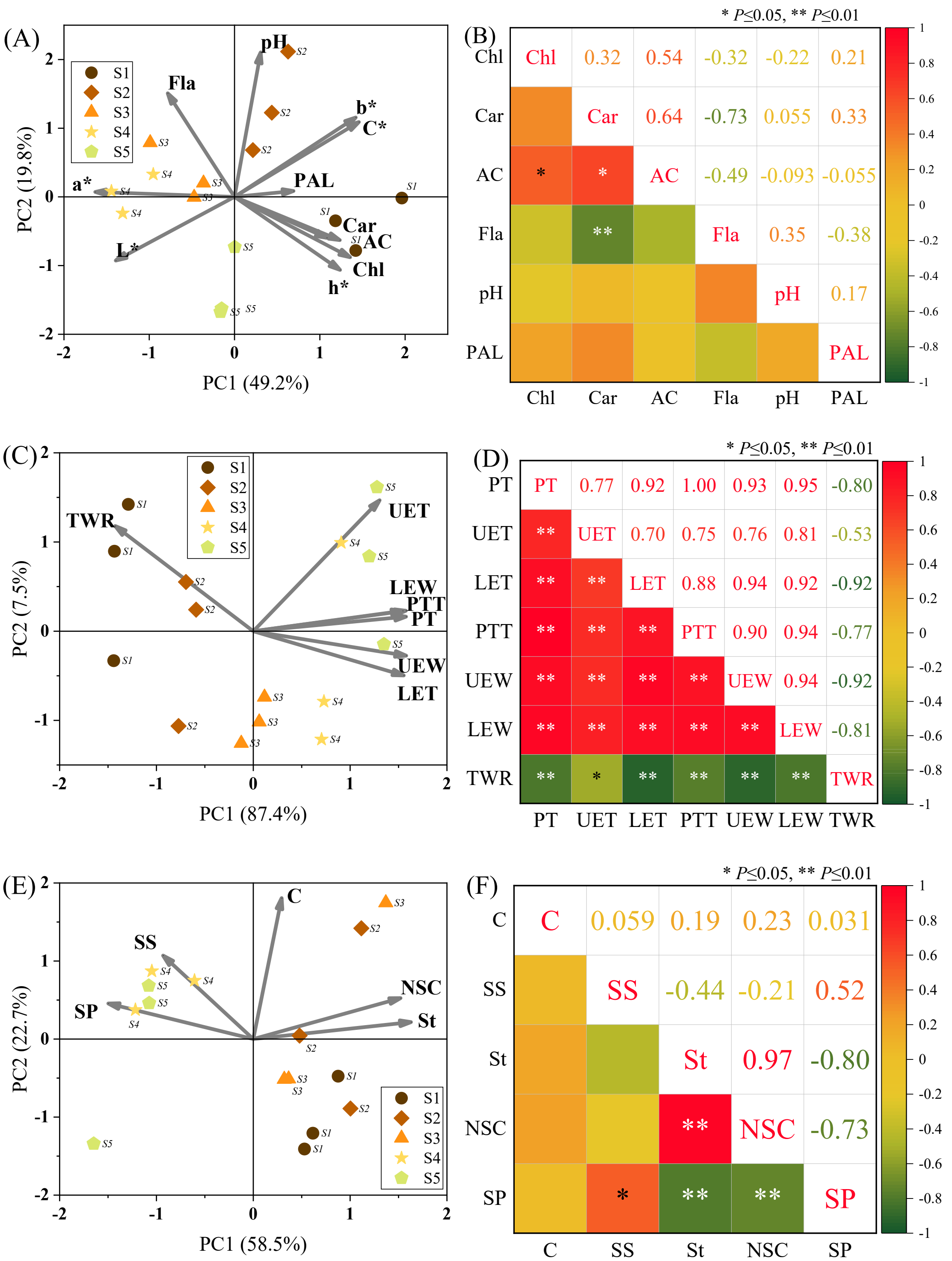

(A)
PC1 (49.2%)
PC2 (19.8%)
S1
S2
S3
S4
S5
pH
Fla
b*
C*
PAL
a*
L*
Car
AC
Chl
h*
(B)
* P≤0.05, ** P≤0.01
Chl 0.32 0.54 -0.32 -0.22 0.21
Car 0.64 -0.73 0.055 0.33
AC -0.49 -0.093 -0.055
Fla 0.35 -0.38
pH 0.17
PAL
Chl Car AC Fla pH PAL
(C)
PC1 (87.4%)
PC2 (7.5%)
TWR
UET
LEW
PTT
PT
UEW
LET
(D)
* P≤0.05, ** P≤0.01
PT 0.77 0.92 1.00 0.93 0.95 -0.80
UET 0.70 0.75 0.76 0.81 -0.53
LET 0.88 0.94 0.92 -0.92
PTT 0.90 0.94 -0.77
UEW 0.94 -0.92
LEW -0.81
TWR
PT UET LET PTT UEW LEW TWR
(E)
PC1 (58.5%)
PC2 (22.7%)
C
SS
SP
NSC
St
(F)
* P≤0.05, ** P≤0.01
C 0.059 0.19 0.23 0.031
SS -0.44 -0.21 0.52
St 0.97 -0.80
NSC -0.73
SP
C SS St NSC SP

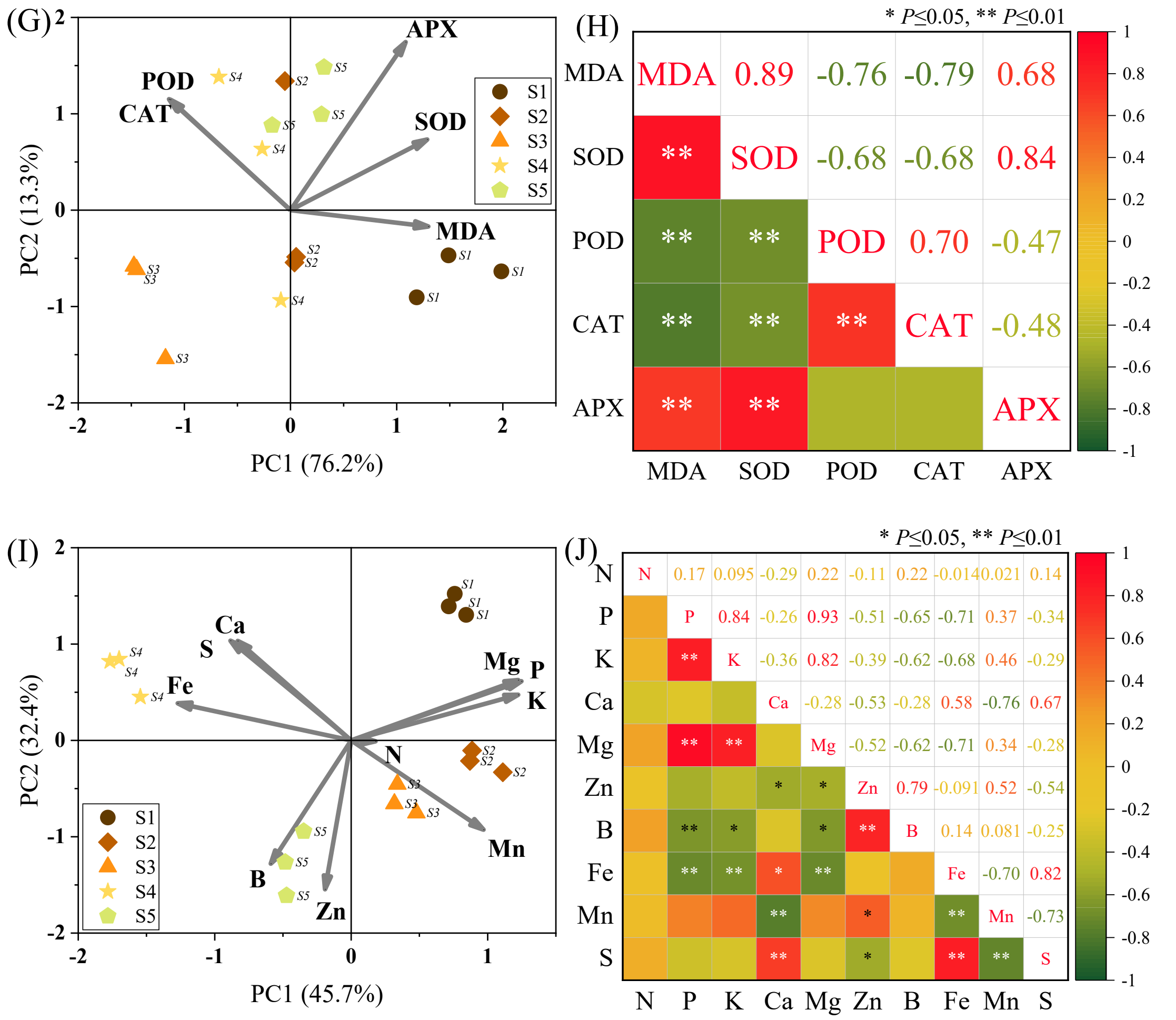

Supplementary 2 PCA and correlation analysis of color parameters and pigments (**A, B**), morphological structure (**C, D**), nutrients (**E, F**), antioxidant activity (**G, H**), and mineral elements (**I, J**) during flower development. PCA was employed to compare the comprehensive changes in indicators across developmental stages. Principal Component 1 (PC1) and Principal Component 2 (PC2) accounted for the first and second highest contribution rates, respectively. Gray arrows represent the measured variables. Arrows pointing in similar directions with acute angles indicate positive correlations between corresponding variables, whereas obtuse angles denote negative correlations. Colored dots correspond to distinct flowering stages; closer proximity of a dot to an arrow indicates a higher value for that specific variable in the respective stage. The correlations in the figure were calculated using Pearson (n=15, 3 biological replicates × 5 stages). * indicates $P \leqslant 0.05$, and ** indicates $P \leqslant 0.01$. Red indicates a positive correlation, while green indicates a negative correlation; the darker the color, the higher the correlation.
