# Supplementary material for: Dynamics of petal pigments and morpho-physiological traits during flower development in Rosa ‘Creamy Eternity’
Source: PeerJ. 2026 Aug 6;14:e21329. doi: 10.7717/peerj.21329 (PMC13453145; doi:10.7717/peerj.21329)
Supplement: Supplementary file 2 [file peerj-14-21329-s001.docx]

 Supplementary 1 – Environmental records

| Date | Maximum Temperature (℃) | Minimum Temperature (℃) | Average Temperature (℃) |
| --- | --- | --- | --- |
| 2025/4/1 | 23 | 13 | 18 |
| 2025/4/2 | 22 | 16 | 19 |
| 2025/4/3 | 22 | 18 | 20 |
| 2025/4/4 | 25 | 19 | 22 |
| 2025/4/5 | 23 | 18 | 20.5 |
| 2025/4/6 | 21 | 19 | 20 |
| 2025/4/7 | 26 | 20 | 23 |
| 2025/4/8 | 24 | 20 | 22 |
| 2025/4/9 | 28 | 21 | 24.5 |
| 2025/4/10 | 31 | 23 | 27 |
| 2025/4/11 | 28 | 22 | 25 |
| 2025/4/12 | 33 | 17 | 25 |
| 2025/4/13 | 26 | 18 | 22 |
| 2025/4/14 | 31 | 18 | 24.5 |
| 2025/4/15 | 35 | 20 | 27.5 |
| 2025/4/16 | 32 | 22 | 27 |
| 2025/4/17 | 29 | 23 | 26 |
| 2025/4/18 | 31 | 24 | 27.5 |
| 2025/4/19 | 34 | 24 | 29 |
| 2025/4/20 | 35 | 24 | 29.5 |
| 2025/4/21 | 35 | 25 | 30 |
| 2025/4/22 | 34 | 23 | 28.5 |
| 2025/4/23 | 33 | 23 | 28 |
| 2025/4/24 | 27 | 21 | 24 |
| 2025/4/25 | 30 | 23 | 26.5 |
| 2025/4/26 | 29 | 23 | 26 |
| 2025/4/27 | 30 | 22 | 26 |
| 2025/4/28 | 30 | 23 | 26.5 |
| 2025/4/29 | 32 | 23 | 27.5 |
| 2025/4/30 | 30 | 21 | 25.5 |
